# Perspectives of Latinx Patients with Diabetes on Teleophthalmology, Artificial Intelligence-Based Image Interpretation, and Virtual Care: A Qualitative Study

**DOI:** 10.1089/tmr.2023.0045

**Published:** 2023-10-20

**Authors:** Christian Pelayo, Johnson Hoang, Maria Mora Pinzón, Loren J. Lock, Christiana Fowlkes, Chloe L. Stevens, Nora A. Jacobson, Roomasa Channa, Yao Liu

**Affiliations:** ^1^ Department of Ophthalmology and Visual Sciences, University of Wisconsin School of Medicine and Public Health, Madison, Wisconsin, USA.; ^2^ Division of Geriatrics and Gerontology, Department of Medicine, University of Wisconsin School of Medicine and Public Health, Madison, Wisconsin, USA.; ^3^ Institute for Clinical and Translational Research, University of Wisconsin School of Medicine and Public Health, Madison, Wisconsin, USA.; ^4^ School of Nursing, Madison, Wisconsin, USA.

**Keywords:** diabetic eye screening, qualitative research, Latinx, virtual care, perspectives

## Abstract

**Background:**

Latinx populations in the United States bear a disproportionate burden of diabetic eye disease. Teleophthalmology with and without artificial intelligence (AI)-based image interpretation are validated methods for diabetic eye screening, but limited literature exists on patient perspectives. This study aimed at understanding the perspectives of Latinx patients with diabetes on teleophthalmology, AI-based image interpretation, and general virtual care to prevent avoidable blindness in this population.

**Methods:**

We conducted semi-structured, individual interviews with 20 Latinx patients with diabetes at an urban, federally qualified health center in Madison, WI. Interviews were transcribed verbatim, professionally translated from Spanish to English, and analyzed using both inductive open coding and deductive coding.

**Results:**

Most participants had no prior experience with teleophthalmology but did have experience with virtual care. Participants expressed a preference for teleophthalmology compared with traditional in-person dilated eye exams but were willing to obtain whichever method of screening was recommended by their primary care clinician. They also strongly preferred having human physician oversight in image review compared with having images interpreted solely using AI. Many participants preferred in-person clinic visits to virtual health care due to the ability to have a more thorough physical exam, as well as for improved non-verbal communication with their clinician.

**Discussion:**

Leveraging primary care providers' recommendations, human oversight of AI-based image interpretation, and improving communication may enhance acceptance and utilization of teleophthalmology, AI, and virtual care by Latinx patients.

**Conclusions:**

Understanding Latinx patient perspectives may contribute toward the development of more effective telemedicine interventions to enhance health equity in Latinx communities.

## Introduction

Latinx populations bear a disproportionate burden from diabetes and have the highest rates of visual impairment and blindness of any ethnic group in the United States.^1–4^ Although early detection and treatment decreases the risk of severe vision loss by 90%, most Latinx patients with diabetic retinopathy remain undiagnosed due to a lack of yearly eye screening as recommended by the American Diabetes Association.^1,5^

As an evidence-based alternative to traditional in-person dilated eye exams, teleophthalmology provides convenient, low-cost screening using retinal photos taken at a primary care clinic.^6,7^ Primary care is an ideal setting for teleophthalmology because nearly 90% of U.S. adults with diabetes regularly obtain care from a primary care clinician.^8^

Teleophthalmology images are usually interpreted remotely by an eye care clinician. More recently, two Food and Drug Administration (FDA)-cleared, autonomous artificial intelligence (AI) algorithms for stand-alone interpretation of images (designed for use without human involvement) have been used to detect diabetic retinopathy from retinal images with high sensitivity and specificity.^9,10^ However, a few recent studies have noted barriers to patient acceptance of AI-based image interpretation.^11,12^

Most studies of Latinx patient perceptions of teleophthalmology and AI use in health care have been survey-based rather than qualitative.^12–14^ The latter provides a richer and more in-depth understanding of complex issues, such as how differences in patient perceptions of new technologies may contribute to health disparities.^12,15,16^

The only two previous qualitative studies evaluating patient perspectives on the use of AI for teleophthalmology were conducted outside the United States and found that while most participants were receptive to the use of AI, a substantial minority expressed a preference for human image review.^15,17^ An improved understanding of patient perspectives may facilitate the development of strategies to effectively integrate AI into telemedicine and enhance health equity in the Latinx community. Thus, our study aimed at understanding Latinx patient perspectives on teleophthalmology, AI-based image interpretation, and virtual care.

## Methods

### Participant recruitment and research setting

Adults (18 years or older) who self-identified as Latinx/Hispanic and diagnosed with type 1 or type 2 diabetes were eligible to participate in semi-structured, individual interviews. A culturally informed recruitment letter and flyer were developed with the following organizations in Dane County, WI: the Latino Health Council of Dane County (a Latinx community stakeholder group), NewBridge Madison (a non-profit organization serving older adults), and Access Community Health Centers (ACHC) (an urban, federally qualified health center).

Of note, this study was conducted before implementing teleophthalmology at ACHC. ACHC staff mailed recruitment letters and flyers to a random sample of their Latinx patients with diabetes (*n* = 523). Patients could opt-in to participate by calling the research team, and recruitment continued until reaching informational redundancy, in which sample size was sufficient so that no new information could be acquired from subsequent interviews.^18^

### Interviews

The interview guide (Supplementary Data S1) was adapted from a prior study among predominantly non-Hispanic, white, rural patients.^19^ A Spanish-language version was created by professional translators and then edited for accuracy by three Spanish speakers on the research team (including a Venezuelan physician with over 10 years of experience in engaging U.S. Latinx communities in research and two Mexican-American research specialists).

Components from the National Institute on Minority Health and Health Disparities (NIMHD) Research Framework were used to create questions that addressed participants' perspectives on teleophthalmology, AI-based image interpretation, and virtual care.^20^ Participants were shown an image of a patient obtaining a traditional dilated eye exam and an image of a patient having their eyes photographed using teleophthalmology (Supplementary Data S2).

Teleophthalmology was described verbally as “a technician uses a special camera to take photos for an eye doctor to look inside your eyes.” To assess their perspectives on AI-based interpretation, participants were asked, “Would you be more comfortable having the eye photo test if the photos were reviewed by an eye doctor or by a computer? Why?”

Demographic information, including age, gender, race and ethnicity, country of heritage or origin, insurance status, diabetes type, duration of diabetes, diabetic eye screening adherence, highest level of education, English fluency, and health literacy using the Single Item Literacy Screener, was collected using a written survey.^21^ The Social Vulnerability Index (SVI), which accesses the vulnerability of communities based on social factors (on a scale 0–1.0, 1.0 being the most vulnerable and 0.46 being the median SVI for the United States overall), was calculated based on a patient's home address zip code using U.S. Census data.^22,23^

From July to November 2022, interviews (45–60 min) were conducted in the participants' preferred language (i.e., either Spanish or English) either virtually or in-person at a community center owned by the University of Wisconsin (UW)-Madison. All interviews were led by a bilingual male Bachelor's-level research specialist (C.P.) with qualitative research training and who self-identifies as Mexican–American. Field notes were taken by the interviewer during the interview. Participants were informed that the interviewer had no specialized medical knowledge of diabetes or diabetic retinopathy and were provided with $30 in compensation for their time and were offered coverage of transportation costs.

### Data analysis

Audio recordings of interviews were transcribed verbatim, professionally translated from Spanish to English, and analyzed using QSR NVivo software for Mac Version 1.7 (QSR International, Melbourne, Australia). We performed inductive open coding and deductive coding using the NIMHD Research Framework and the Campbell and Egede Model, which describes challenges experienced by inner-city African-Americans in managing type 2 diabetes.^20,24^

The research specialist (C.P.) performed independent open coding of the first five transcripts. The research team members, N.J. (a PhD qualitative methodologist), and members of the UW Institute of Clinical and Translational Research-Community Academic Partnership (ICTR-CAP) Qualitative Research Group iteratively reviewed codes and refined the coding framework. The Principal investigator (Y.L.) ensured consistency by dual-coding every fifth transcript.

To enhance rigor, we performed member-checking to review our results with a subset of participants and community stakeholders (i.e., patients [*n* = 3] and Latinx community members recruited from NewBridge Madison [*n* = 3]) in two separate 1-h meetings.^25^ Members judged our interpretation of the interview data to be accurate and complete and provided more nuanced perspectives. Our report of this study follows the Consolidated Criteria for Reporting Qualitative Research (Supplementary Data S3).^26^

### Ethics/Institutional Review Board review

The UW-Madison Health Sciences Institutional Review Board (IRB) determined that this interview research met criteria for exemption. Following the guidance of the IRB, the interviewer (C.P.) obtained verbal consent from all participants. All research activities were conducted in accordance with the Declaration of Helsinki.

## Results

### Participant characteristics

Among the 22 Latinx individuals with diabetes who contacted the research team in response to the recruitment flyer, two did not attend an interview after multiple rescheduling attempts. Interview participants (*n* = 20) all had a diagnosis of type 2 diabetes and had a mean age of 59.8 years (range: 33–79 years) (Table 1). Most participants were female (60%, *n* = 12), reported either Mexican origin or heritage (60%, *n* = 12), were uninsured (60%, *n* = 12), and had low or moderate health literacy (60%, *n* = 12).

**Table 1.** Demographics of Patient Interview Participants (*n* = 20)

Characteristics	Mean (SD) (***n***) (range) or % (***n***)
Age (years)	59.8 (14.0) (20) (range: 33–79)
Female	60% (12)
Latinx/Hispanic	100% (20)
Country of origin or heritage	
Mexico	60% (12)
Bolivia	10% (2)
Venezuela	10% (2)
Argentina	5% (1)
Colombia	5% (1)
Dual origin (Mexico-USA and Puerto Rico-El Salvador)	10% (2)
Type 2 diabetes	100% (20)
Duration of diabetes	
<5 years	20% (4)
5–10 years	35% (7)
10–15 years	20% (4)
15+ years	25% (5)
Non-adherence with diabetic eye screening	70% (14)
Prior experience with teleophthalmology	10% (2)
Prior experience with virtual care	95% (19)
Uninsured	60% (12)
SVI^23^	0.59 (0.28) (20) (range: 0.04–0.98)
Education	
Elementary school or less	15% (3)
Middle school graduate	20% (4)
Some high school	5% (1)
High school graduate or GED	25% (5)
Some college or technical school	15% (3)
College graduate	20% (4)
Health literacy—single-item literacy screener^30^	
High	40% (8)
Moderate	35% (7)
Low	25% (5)
Primary language spoken at home	
Spanish	80% (16)
English	10% (2)
Both	10% (2)
Preferred language in clinic	
Spanish	75% (15)
English	15% (3)
Both	10% (2)
English fluency	
Not at all	15% (3)
Not very well	40% (8)
Well (conversational)	15% (3)
Very well (fluent)	30% (6)
Who helps you at your clinic visits if you do not speak English fluently? (*n* = 16)	
Interpreter	94% (15)
Self	6% (1)
Usual mode of transportation to clinic	
Car—self	70% (14)
Car—family	10% (2)
Bus	5% (1)
Insurance-provided transportation	5% (1)
Multiple methods (family, taxi or insurance-provided)	10% (2)

GED, general educational development; SD, standard deviation; SVI, Social Vulnerability Index.

More than half had an English fluency of “not at all” or “not very well” (55%, *n* = 11), and most preferred to speak Spanish during their clinic appointments (75%, *n* = 15). The majority (70%, *n* = 14) had not received diabetic eye screening within the past year, and only two participants (10%) had eye photos taken in the past. Nearly all had prior experience with virtual care (95%, *n* = 19). Interviews were primarily conducted in-person (90%, *n* = 18) and in Spanish (85%, *n* = 17).

Two participants (10%) were acquainted with the interviewer (C.P.) from his prior work on increasing COVID-19 vaccination rates in the Latinx community in partnership with the Latino Health Council of Dane County.

### Teleophthalmology versus traditional in-person dilated eye exam for diabetic eye screening

Many participants reported preferring teleophthalmology over a traditional in-person dilated eye exam, because they believed that teleophthalmology represented more advanced technology that had greater precision and accuracy (Table 2). One participant specifically noted a general perception that electronic equipment (i.e., teleophthalmology) worked better than manual processes, such as an in-person dilated eye exam.

**Table 2.** Teleophthalmology Versus Traditional In-Person Dilated Eye Exam for Diabetic Eye Screening

Themes	Example quotes
Teleophthalmology is better at detecting eye disease than an in-person eye exam	“[Teleophthalmology] is advanced technology and can point out things that the old test can't or find things that the old test can't or is more accurate.” (Patient 6) “It seems to me that electronic equipment works better than manual things like [a traditional dilated eye exam].” (Patient 10) “I suppose [teleophthalmology is] more precise because science is advancing, not the other way ‘round [*laughs*]… I think I'd rather have the most advanced [screening method] because it can help me more.” (Patient 14)
Teleophthalmology allows the patient to see their screening results with the eye photo	[The patient] can see what [their] eye looks like—what's there, which you can't see with the [traditional eye exam].” (Patient 1) “With the photo, we can compare this photo… to another photo next year or in two years, in three years. And I don't have to rely on my memory or my notes to see if there's a difference.” (Patient 18)
Teleophthalmology doesn't require dilating eye drops	“[For teleophthalmology…] you don't need drops… and to find someone who can drive… when it's over.” (Patient 15)
Teleophthalmology is more affordable, convenient, and time efficient	“$20 [for an eye photo] isn't that bad either, though. It's really not. That's very, very cheap, because I'm assuming this [camera that takes the eye photo] is probably thousands of dollars.” (Patient 5) “[I prefer teleophthalmology] because it's more efficient… I've been sent to [a different clinic] for [eye] tests, so, it would be nice if [the primary care clinic] had their [own] eye clinic as well.” (Patient 1) “If [my primary care clinic] gave me a yearly eye exam, I'd be happy because it would give me peace of mind knowing that I can see my progress every year.” (Patient 2)
Teleophthalmology complements the in-person physical exam by the PCP^a^	“After [the PCP] examines you, they can see in the picture if there is or isn't any damage… [in case the PCP] couldn't see it, huh? So, I think one complements the other, but the [PCP's] examination is very important.” (Patient 7)
Distrust of technology	“I feel like [people would prefer] the standard one… Many people don't really trust technology that much.” (Patient 8)
Preference for following a doctor's recommendation regarding method of diabetic eye screening	“I trust the doctors and whichever [method of eye screening] that they say that I should do, that's the one I'm going to do. Because they're the ones who are going to read the results, and what they think is going to be best.” (Patient 2) “[It's] not for me to decide [which exam to get]. That's for the doctor to decide… and I'd agree.” (Patient 10) “You have to follow everything [the doctor] says… You can't say ‘No, the drops make my eyes hurt’… The doctor knows what they're doing. They're specialists.” (Patient 12) “I wouldn't know how to answer [which type of screening I would prefer], because… only those who do eye exams know how to check it… I trust them, because that's what they study, and they are qualified.” (Patient 19)

^a^ PCP, primary care clinician.

Participants also noted several logistical benefits of teleophthalmology, including that it does not require dilating eye drops that blur their vision and require them to have a driver. Further, most participants felt that offering teleophthalmology for an out-of-pocket cost of $20 (i.e., amount equivalent to standard co-pays for in-person clinic visits, such as for dilated eye exams, among insured patients) would be acceptable and that teleophthalmology would be more time-efficient than an in-person dilated eye exam.

Participants also noted the benefits of having teleophthalmology eye photos as part of their medical records. Some appreciated the opportunity to directly review their results by seeing their eye photo. Another perceived benefit was that teleophthalmology allows for the comparison of eye photos over time to detect changes. In addition, some participants believed that teleophthalmology complements the eye exam performed by a primary care clinician to catch diabetic eye disease that might otherwise be missed.

While most participants were highly receptive to teleophthalmology, a few participants indicated distrust of new technologies and preferred an in-person dilated eye exam. Yet, all participants reported that they would obtain whichever type of diabetic eye screening was recommended to them by their primary care or eye care clinician. Participants expressed their belief that doctors are the most knowledgeable as to which type of screening would be most appropriate for an individual patient and emphasized their trust in the expertise of health care professionals.

### AI versus physician-based image interpretation of teleophthalmology

Participants strongly preferred that a human physician be involved in the review and interpretation of their eye photos, rather than relying solely on an AI algorithm (Table 3). Many reported trusting the education and professional experience of a human physician, because they valued the human capacity to learn and reason when encountering unfamiliar scenarios, in contrast to “a machine does what it's already programmed to do, nothing more” (Patient #13).

**Table 3.** Artificial Intelligence Versus Physician-Based Diagnostic Interpretation of Teleophthalmology for Diabetic Eye Screening

Themes	Example quotes
Preference for a human physician to review the eye photos vs. AI	“[I would prefer] a physician… because they're going to review [the eye photos] more thoroughly… Although, I don't even know how advanced the computer is.” (Patient 4) “I'd want the doctor. Because computers make mistakes, I mean, human beings definitely make mistakes, but I trust the doctor because he's educated, he's gone to medical school for some years, probably got a reputation. And the computer does make mistakes, it doesn't have a reputation. It hasn't gone to school.” (Patient 5) “The ophthalmologist. Because he is better, right? Because he has more experience. There's also the human part involved… Even if there's a machine, the machine does what it's already programmed to do, nothing more… Doctors always put all their mental capacity, what they've learned, emotions or that way of seeing things honestly… (Patient 13) “I think that the ophthalmologist has more experience, since they've been working for several years… Although the computer is more accurate, doctors have the experience of being there… I feel safer when a person sees it.” (Patient 20)
Preference for a human physician to use AI for decision support, but make the final diagnosis	“I will feel extremely comfortable having a computer analyze and compare [my eye photos] with millions of other pictures… in a fast way… But I also want to make sure that a person who will be treating me and giving me advice sees that [result]… and makes their own decision and is not a machine. So, the machine can inform the doctor.” (Patient 18)
Ambivalence toward AI-based eye screening	“I think that if [teleophthalmology results are] checked by a computer, it's better than by a doctor… because we're at a level where computers are perfect… I don't know, it's difficult because human beings have something that a computer will never have, which is feelings.” (Patient 1) “I trust human beings more than machines. Although, computers are precise.” (Patient 3)

AI, artificial intelligence.

Further, some participants noted that human physicians are motivated to help patients due to having empathy, as well as to avoid making mistakes that could harm their professional reputation. In addition, participants expressed safety concerns that computers could make serious mistakes, and some noted uncertainty regarding the accuracy of AI.

Most participants reported feeling comfortable with the use of AI as a decision support tool for human physicians. Even when they acknowledged the benefits of AI in providing a fast and accurate analysis that may exceed that of a human physician, many participants still wanted the human physician involved in the final decision-making process because of their greater trust in health care professionals compared with machines.

### Experiences with and perspectives on virtual health care

Participants reported that they appreciated the convenience and time efficiency of virtual health care (i.e., not specific to eye care), which removes important barriers to access (Table 4). One participant found virtual appointments over the phone particularly helpful when struggling with depression and having trouble just getting out of bed.

**Table 4.** Experiences with and Perspectives on Virtual Health Care

Themes	Example quotes
Virtual care removes barriers to care due to being more convenient and time efficient	“I liked [the virtual visits]. And they were good for me because I struggle with depression really bad and sometimes, I can't even get out of bed. And when you don't want to get out of bed and you don't want to deal with the world, it's nice to have that appointment over the phone.” (Patient 5) “Sometimes [virtual care is] okay because sometimes you don't have that much time… to go to your appointment.” (Patient 8) “So, for regular care… maybe the annual checkups or new diagnosis or stuff like that, I think that I like the in-person a lot better. If it's just diagnosis, medication… I think telehealth is extremely helpful. It's fast, and it gives you that.” (Patient 18)
Inability to adequately assess body language during a virtual care encounter	“[Virtually] I can read people's body language… I have a much better time because I can make good choices for myself, who's good for me and who's not. But over the computer I can't do that.” (Patient 6) “When you're present, the body language tells you that, ‘Yes…’ you have another problem, and the doctor would ask, ‘Does something hurt?’ or whatever. But you can't see that by videoconference.” (Patient 10)
Virtual care isn't adequate when a patient needs a physical exam	“Let's say I call to tell [a doctor] that I feel sick and nauseous and that I'm vomiting, I'm [willing to follow their instructions]. But if you're going to make a call because you need the doctor to auscultate you, I don't see how [that's possible].” (Patient 1) “I think that in-person [care] is better, because if there's a problem, according to my symptoms, if I feel bad, the doctor can refer me to have my blood sugar tests done to check my levels or take my [blood] pressure. I have a device [at home], but I don't know if it's okay.” (Patient 16)
Virtual care should be used only when it is not possible to obtain in-person care	“It would be better [to obtain care] in person, but if it can't be done in person, only by phone, then, there's no other way.” (Patient 13) “[Virtual care] was strange because I usually always have the appointment… in person, but there was no other way, so [virtual care] was better than nothing.” (Patient 14)
Discomfort with virtual care due to lack of experience with using it	“[I wouldn't feel comfortable with virtual care] since I've never done it, I always go to see [my doctor] in person, so for me, I'd like it better if [my care was] in person.” (Patient 9)
Importance of in-person care for establishing an emotional connection with the doctor	“I'd prefer a face-to-face [in-person] visit because there's nothing better than having a person there, you know? Because you see emotions in person. It's as if you kind of connect with that person.” (Patient 2) “In the middle of the pandemic, when… my wife [died], [my primary care clinician] hugged me in the middle of the pandemic when the COVID situation was at its peak. And that proves that—I mean, the human warmth of the doctor, right? [That is] exactly [what I would miss].” (Patient 3)

Participants also appreciated the time-saving aspects of virtual care, particularly for shorter appointment types, such as those for medication management that may not require a physical exam. However, most participants preferred in-person visits for their annual physical exams or to evaluate symptoms that could be related to a new diagnosis.

Some participants felt that virtual care is inadequate when they believe a physical exam is needed to fully evaluate their medical problem. In addition, there was a preference for in-person visits when they must go to the clinic to get laboratory tests done, such as for hemoglobin A1c. Likewise, despite having a home blood pressure monitor, another participant reported feeling more confident in blood pressure measurements obtained at a clinic.

Many participants also emphasized the importance of in-person care for establishing an emotional connection with a physician, because they felt that they could detect emotions more clearly. One participant specifically mentioned the importance of having support from human warmth and physical contact with their doctor, particularly during difficult times.

Some participants also expressed concerns about the inability to fully assess a clinician's body language during virtual care encounters that would help them better assess whether to trust that clinician and follow their recommendations. Conversely, one participant noted that a patient's body language can also help clinicians identify additional problems that patients may not verbally express.

Another participant reported feeling uncomfortable with virtual care and had never used it. Some participants suggested that virtual care should be used only when in-person care is not possible.

## Discussion

In this qualitative study, most participants perceived teleophthalmology to be superior to in-person dilated eye exams for diabetic eye screening because they felt that it was advanced technology and had better accuracy. In addition, they preferred teleophthalmology because it was offered at an acceptable cost, was more time-efficient, was conveniently located in the primary care clinic, and did not require the use of dilating eye drops. In contrast, a small number of participants expressed a distrust of technology and preferred an in-person eye exam. All participants reported that they would follow their physician's recommendation as to which screening method would be most appropriate for them. Further, most participants preferred to have oversight from a human physician in reviewing their eye photos rather than evaluation solely using AI. While participants regarded virtual health care to be more accessible and efficient, they noted that virtual visits might be inadequate for medical issues that required a physical exam, and that it was more difficult to establish an emotional connection with their clinician compared with an in-person clinic visit.

Our qualitative study builds upon the limited existing literature on perceptions of teleophthalmology in Latinx communities. A focus group study among Latinx and Black patients undergoing teleophthalmology found that many did not understand its purpose, how the program worked, how to receive their results, and what to do after the test.^27^ Fear of technology was voiced by some of their participants. Yet, all participants in this prior study had used teleophthalmology despite significant knowledge gaps and concerns, which suggests that factors not described in their study were able to overcome these barriers (possibly a recommendation from their primary care clinician, which was a strong facilitator noted by participants in our study). Another study among predominantly Latinx adults found that 88% would highly recommend teleophthalmology to others and appreciated having the camera conveniently located in a community center.^28^

Our results agreed with these previously reported findings in that most Latinx individuals generally held positive views of teleophthalmology and providing the service in a convenient location was highly valued. However, a limitation of the community-center based study was that 85% of the participants did not have a diagnosis of diabetes and thus would not be eligible for teleophthalmology in health care settings.^28^ Further, our study identified that the perceived accuracy of teleophthalmology contributes toward positive Latinx attitudes toward this technology.

Prior studies regarding non-Latinx patient perceptions of AI-based interpretation of teleophthalmology found high acceptance of this technology. An Australian study among patients who had experience with both human and AI-based image interpretation for teleophthalmology found that 96% of participants were either satisfied or very satisfied with AI-based interpretation, and 78% preferred AI-based interpretation.^17^ A New Zealand study also found that most respondents who were obtaining teleophthalmology screening reported that they were comfortable with the use of AI in their care, but only about half reported trusting AI as much as a human health care professional.^15^ Perceived benefits of using AI included faster diagnostic speeds and greater accuracy, but 36% still preferred human image interpretation even though it would take longer to receive their screening results.

In contrast, we found that most of our participants preferred to have humans involved in teleophthalmology image review. Concerns about cybersecurity were also raised in these prior studies but were not mentioned by participants in our study. Notably, neither of these prior studies reported the inclusion of any Latinx patients, whereas our study specifically focused on Latinx patient perspectives.

Patient perspectives on the use of AI in health care have found variability in its acceptance based on race and ethnicity, as well as health care application. A survey study by Tyson et al. found that Latinx patients were more likely to accept the use of AI for skin cancer screening (69%) compared with White (65%) and Black (57%) respondents.^12^ In contrast, 40% or fewer of all U.S. adults reported accepting AI for pain management after surgery, AI-based surgical robots, or AI-based mental health chatbots. Our study found that most Latinx patients preferred human oversight in AI-based interpretation for teleophthalmology because they valued physicians' education, experience, empathy, ability to reason in unfamiliar scenarios, and professional accountability.

Our study also expanded the literature on Latinx perspectives regarding virtual care. A prior study found that Latinx participants expressed some concerns about confidentiality, privacy, and the physical absence of the clinician in virtual care interactions, but to a lesser extent than African Americans.^13^ In our study, Latinx patients reported the inability to assess body language as an important limitation of virtual care because of their concern that a patient's ability to decide whether to trust a clinician was diminished.

Another study found that Latinx parents significantly preferred in-person rather than virtual visits for their children as compared with non-Latinx parents (61.3% vs. 28.6%).^14^ The inability to conduct a thorough physical exam was viewed as a significant limitation of virtual care, particularly for a new or potentially serious diagnosis.

A pre-existing positive relationship with the clinician made the use of virtual care more acceptable. An additional theme noted by Latinx participants in our study included concerns that virtual care limited one's non-verbal communication and emotional connection with a clinician.

Of note, our interview participants may have had virtual care experiences with providers who had limited training in how to strengthen patient-provider relationships in telehealth and/or a few protocols in place due to the need to rapidly adopt virtual care models at the time of the COVID-19 pandemic. If providers had the benefit of such training and protocols, then it is possible that participants may have felt more of an emotional connection with their virtual care providers.

Possible solutions include providing hands-on training to health care providers on conducting a 3-way virtual call with an interpreter, properly orienting the patient to the telehealth visit, making eye contact by looking into the camera, providing a clear view of one's face and body language for gestures, affirmational head nodding and smiling, talking about non-health care-specific subjects to establish a social connection, demonstrating active listening, and the use of a validated checklist such as the Teaching Interpersonal Skills for Telehealth Checklist.^29,30^

While our study had several strengths, such as recruiting a large proportion of uninsured participants and those non-adherent with screening (populations less likely to participate in research), it also had a few limitations. Our study seeks to assess knowledge, beliefs, and acceptability of teleophthalmology and AI-based image interpretation among Latinx patients, the majority of whom did not have firsthand experience with these technologies.

We acknowledge that patients' perspectives might change if they did have such experiences. For example, patients may find that the advantage of having much more rapid receipt of results at the point-of-care from AI-based image interpretation outweighs their perceived desire for human involvement in image interpretation, as was noted by a few participants in an Australian study of non-Latinx patient populations.^17^ In addition, the two current FDA-cleared AI algorithms for teleophthalmology interpretation are specifically designed to be autonomous for stand-alone interpretation of medical images without human involvement. Thus, we investigated in this study how Latinx patients with diabetes perceived the use of this currently available AI technology. It would be interesting in future studies to assess patients' perceptions on varying methods for using AI in conjunction with clinicians in teleophthalmology image interpretation.

Other limitations of our study include that the “opt in” method of recruitment requiring patients to call in to the research team to participate likely selected for a more motivated patient population than a study that used an “opt out” method of recruitment in which researchers may directly contact any patient who did not explicitly “opt out” from contact by researchers. Participants were predominantly Mexican in origin and heritage, consistent with the demographics of Latinx populations in Wisconsin.^31^ To assess the generalizability of our findings, future studies could consider using “opt out” recruitment methods, as well as include Latinx patients who have had teleophthalmology imaging, AI-based image interpretation, those living in other regions of the United States, and those with other national origins or heritage.

In summary, we found that Latinx participants preferred teleophthalmology to in-person dilated eye exams, strongly preferred human oversight of AI-based retinal image interpretation, and had concerns about the effectiveness of non-verbal communication in virtual care visits. Our qualitative data allow us to better understand the reasons that underlie Latinx patient perceptions that may contribute to the development of more effective telemedicine interventions to enhance health equity in Latinx communities.

## Supplementary Material

Supplemental data

Supplemental data

Supplemental data

## Abbreviations Used

ACHC: Access Community Health Centers
AI: artificial intelligence
CTSA: Clinical and Translational Science Award
DSMC: Data and Safety Monitoring Committee
FDA: Food and Drug Administration
GED: general educational development
I-TRUST: Implementation of Teleophthalmology in Rural HealthSystems
ICTR-CAP: Institute of Clinical and Translational Research-CommunityAcademic Partnership
IRB: Institutional Review Board
NCATS: National Center for Advancing Translational Sciences
NEI: National Eye Institute
NIH: National Institutes of Health
NIMHD: National Institute on Minority Health and Health Disparities
PCP: primary care clinician
SD: standard deviation
SVI: Social Vulnerability Index
UW: University of Wisconsin
