# Supplementary material for: Perspectives of Latinx Patients with Diabetes on Teleophthalmology, Artificial Intelligence-Based Image Interpretation, and Virtual Care: A Qualitative Study
Source: Telemed Rep. 2023 Oct 20;4(1):317–26. doi: 10.1089/tmr.2023.0045 (PMC10615055; doi:10.1089/tmr.2023.0045)
Supplement: Supplemental data [file Suppl_DataS1.pdf]

Introduction:

Thank you so much for taking the time to meet with me. Our research team at the University of Wisconsin is working with Access Community Health Centers to learn more about patient experiences with diabetic eye checks and communicating with healthcare providers.

We hope to use the knowledge we gain from these interviews to understand how to prevent vision loss from diabetes by learning what makes it easier or harder to get diabetic eye checks for you and others in your community.

You were invited to participate because you receive care for diabetes at Access Community Health Centers. I expect our conversation will last about an hour. At the end of our discussion, I will ask a few questions about your background.

Participation in this interview is voluntary. Your decision whether to participate will not affect your care at Access Community Health Centers. You can stop the interview at any time and if there are any questions you don't want to answer you can just tell me to skip those.

Everything you tell me today will be kept confidential. Only our research team, led by Dr. Maria Mora Pinzón, a University of Wisconsin preventive medicine doctor, and Dr. Yao Liu, a University of Wisconsin eye doctor, and will have access to this information. I am a research team member working with them and while we will be discussing health-related topics, I want to let you know that I do not have a medical background.

Protected health information (PHI), is information about your physical or mental health that includes your name or other information that can identify you. To do this study, we will use things you tell the research team about your health.

Your authorization for researchers to use your PHI does not have an end date. However, you can choose to take back your authorization for researchers to use your health information. You can do this at any time before or during your participation in the research. If you take back your authorization, you will not be able to take part in the research study. To take back your authorization, you will need to notify the researchers.

The information collected in this study, including your health information, may be used or shared for future related research.

Audio recordings will be made of the interview so that I can review our discussion later and make sure I accurately get all the ideas and opinions that you share. Only the researchers will have access to these recordings. The researchers or someone hired by the researchers will listen to the recording and write down what people said during the interview. The written copy is called a transcription. The transcription will be saved but the recording will be destroyed. No information that could identify you will be included in the transcription.

As a reminder, this interview is about your personal experiences. There are no "right" or "wrong" answers.

If you think of any questions as we go along, please feel free to ask them any time. Do you have any questions before we begin?

Patient Interview Guide – Patients with eye screening

Part I. General Questions About Diabetic Eye Checks

First, I'd like to ask you some general questions about how you take care of your diabetes.

Can you walk me through the things you do each day to take care of your diabetes?

Prompts:

□ *[if none]* What are some of the things your doctor may have discussed with you that may be important for people with diabetes to do?

You have probably heard that diabetes can affect our eyes and vision.

What are some things that people with diabetes can do to keep their eyes healthy?

Prompts:

□ Have you heard of any foods, supplements or herbs that might help to keep your eyes healthy?

□ What information have you heard about getting regular eye checks for people with diabetes?

If someone with diabetes has worsening vision, what can they do to prevent their vision from getting worse?

Prompts:

□ Would going to see an eye doctor be helpful? If so, what might the eye doctor do to help?

Now I'd like to show you some pictures. These photos show two ways of doing eye checks for people with diabetes. The top photo shows a Traditional Eye Exam, where an eye doctor uses eye drops to open up your eyes so that they can see inside. The bottom photo shows an Eye Photo Test, where a technician uses a special camera to take photos for an eye doctor to look inside your eyes.

Some people get yearly eye checks as part of their diabetes care. What experiences have you had with diabetic eye checks?

Prompts:

□ How do you know when it is time for you to get an eye check for diabetes?

□ Why did you choose to get your eyes checked?

Where did you learn about getting diabetic eye checks?

Prompts:

□ *[If they don't currently get information about eye exams.]* Where did you learn about how to take care of your diabetes?

□ What do you know about diabetes programs available through Access Community Health Centers?

□ From where in your community might you want to learn more about diabetic eye checks? *[or how would you like to learn about diabetic eye checks?]*

□ Would you like to receive information about taking care or your diabetes in a written brochure, watch a video, meet with a group or meet individually with a diabetes educator?

Would you get a diabetic eye check if it was recommended by your regular doctor who takes care of your diabetes? Why or why not?

Prompts:

□ How would you decide whether to get a diabetic eye check?

□ If you needed to pay to get a diabetic eye check, how much would you be willing to pay?

□ If you have insurance, does your insurance cover Eye Exams?

In your experience, how easy is it to regularly get diabetic eye checks as compared to *[an example the participant provided about what they do for their diabetes, preferably a clinic test such as getting a lab draw to check hemoglobin A1c]***?**

Prompts:

□ Can you tell me more about why you find it easier/harder?

□ You mentioned [x] as one thing that makes it hard to regularly get eye exams. Can you tell me more about that? What else might make it hard?

□ *[if cost or finances are mentioned]* You mentioned that cost can be an issue. How does cost affect your decision to get your eyes checked?

What do you think would help more people with diabetes in your community to get their eyes checked?

[If patient has not had teleophthalmology, jump to page 6: Version II. Clinical Eye Exam]

Version I. Teleophthalmology

I believe you had the Eye Photo Test *[point to photo]* ***[x]*** **weeks/months ago, is this correct?**

Tell me about your experience starting with how you first learned about the Eye Photo Test.

Prompts:

□ Do you remember who explained this test to you?
□ What did they tell you about it?
□ Did you get all the information you wanted?
□ What made you decide to have the Eye Photo Test?
□ Why do you think your doctor referred you for the Eye Photo Test?

Next, I have a few questions about scheduling the Eye Photo Test and getting to your appointment.

How easy was it to schedule the Eye Photo Test?

Prompts:

□ How long did you wait between the time you scheduled the Eye Photo Test and when you had the appointment? (e.g. a few days, weeks or months)
□ How easy was it to get to the clinic for the photo eye test? Did you drive yourself or did someone else drive you? How long did it take to get from your home to the clinic?

Now I'd like you to tell me about your experience with the Eye Photo Test itself.

Prompts:

□ About how long did the Eye Photo Test take?
□ Did you receive results from your Eye Photo Test?
□ [If YES] How quickly did you get those results? Is there anything that could be changed or improved about the way you receive the results?
□ [If NO] Did you want to receive the results from the Eye Photo Test?
□ How would you like to receive your Eye Photo Test results? (e.g. letter in the mail or phone call)

How would you explain the Eye Photo Test to a friend or family member?

Prompts:

□ Would you be willing to take the Eye Photo Test again? Why or why not?

Do you think that there is anything that could be improved about your experience with the Eye Photo Test?

Prompts:

□ How can we make the Eye Photo Test more available to other people in your community who have diabetes?

□ For your community, do you think $20 is a reasonable cost for the Eye Photo Test?

Do you plan to have another diabetic eye check in the future?

Prompts:

□ [If YES] How soon and where might you schedule this? [verify what type of exam – Traditional Eye Exam vs Eye Photo Test]

□ [If NO] Why not?

Let's look at the picture of the Traditional Eye Exam [point to photo]. Have you ever had this exam where an eye doctor uses eye drops to dilate and examine your eyes?

Prompts:

□ [If YES] Tell me about your experience with this exam. What was it like?

Let's look at the picture of the two types of eye checks again. If you had to choose between having your eyes checked using a Traditional Eye Exam or an Eye Photo Test, which would you prefer? Why?

Prompts:

□ What might be some reasons why someone would prefer *[the other option: the traditional eye exam OR the eye photo test]* instead?

□ Would you be more comfortable having the eye photo test if the photos were reviewed by an eye doctor or by a computer?

Is there anything else you think is important for me to know about your experiences with getting diabetic eye checks?

[Jump to Part II on page 7]

Version II. Clinical Eye Exam

I believe you have not had the Eye Photo Test *[point to photo]*, is this correct?

Now, let's look at the picture of the Traditional Eye Exam *[point to photo]*. Do you recall roughly when was the last time you had a Traditional Eye Exam?

How would you describe the Traditional Eye Exam to a friend or family member?

Prompts:

□ Would you be willing to have a Traditional Eye Exam again? Why or why not?

□ *[IF NO]* What would have to change for you to choose to get your eyes checked?

Does your insurance cover Traditional Eye Exams? How much do you normally pay to have a Traditional Eye Exam?

If you had to choose between having your eyes examined using a Traditional Eye Exam or an Eye Photo Test, how would you decide which test to have? Which test might you prefer? Why?

Prompts:

□ What might be some reasons why someone would prefer *[the other option: the traditional eye exam OR the eye photo test]* instead?

□ Would you be more comfortable having the eye photo test if the photos were reviewed by an eye doctor or by a computer?

Is there anything else you think is important for me to know about your experiences with getting diabetic eye checks?

Part II. Questions Related to Communication Preferences

Thanks so much for this very helpful information. To gain a more complete understanding of your experiences, some of the topics we would like to discuss today relate to where you choose to obtain healthcare, as well as your perspectives and experiences communicating with healthcare providers.

How did you decide to go to your current medical clinic for healthcare?

Prompts:

□ How important is it for you to find a healthcare provider that speaks [Spanish or preferred language]?

□ Does your family help you decide where to go for healthcare? If so, how do they help?

[If patient seems to be comfortable with where they get their healthcare]
When I listen to you, I get the sense that you are quite comfortable with getting healthcare [at *[X clinic/healthcare organization] or with Dr. X]*. Is that right? Can you talk with me about things that made you feel comfortable at that [clinic/place]?

What might make you feel more comfortable with getting care at an eye clinic where you may not have been to before?

Prompts:
□ How do you find information about where you might want to go to get your eyes checked?
□ Who in your community would you reach out to for a recommendation about an eye clinic or eye doctor?
□ What could an eye clinic do to make you feel more comfortable getting your care there?

Now, I want to think of a time when you a very good discussion with your doctor or nurse about your health. Please tell me what made that discussion a good experience.

Prompts:
[IF THEY HAVE AN EXAMPLE]
□ What language did you use? Did you use an interpreter?
□ What made you feel more comfortable asking questions?
□ What made you feel less comfortable asking questions?
□ What questions did you have that didn't get answered due to a difficulty with communication?

[IF THEY DON'T HAVE AN EXAMPLE]
Please think of a discussion with your doctor or nurse about your health that could have gone better. What could have made the discussion go better?
□ What language did you use? Did you use an interpreter?
□ What made you feel more comfortable asking questions?
□ What made you feel less comfortable asking questions?
□ What questions did you have that didn't get answered due to a difficulty with communication?

Now I'd like to discuss any experiences you have had with using telemedicine. During the COVID-19 pandemic, some patients with diabetes have chosen to have video or phone telemedicine visits with their doctors instead seeing them in-person at the clinic.

What experience have you had with using video or phone telemedicine visits with any of your doctors?

Prompts:

□ *[IF HAS EXPERIENCE WITH TELEMEDICINE]* When did you start having these types of visits?

- □ How comfortable do you feel with video or phone telemedicine visits with your doctors?
- □ What do you like about these types of visits compared to seeing your doctor in-person?
- □ What do you *dis*like about these types of visits compared to seeing your doctor in-person?

□ *[IF DOES NOT HAVE EXPERIENCE WITH TELEMEDICINE]* How comfortable would you feel with video or phone telemedicine visits with your doctors?

- □ How do you feel about these types of telemedicine visits compared to seeing your doctor in-person?

[stop audio recording here]

Part 3. Wrap-up and Demographic Survey

Is there anything else you think is important for me to know about diabetic eye checks or communicating with healthcare providers that I haven't asked about?

Thanks so much for all this great information. I really appreciate you sharing your experiences with me. Our goal is to use the knowledge we gain from these interviews to improve eye care services for people with diabetes in your community.

There is just one last thing—a short list of questions to get some background information. Please understand that we only want this information so that we have an accurate picture of who is involved in our study; we know the community is diverse and we want to be sure we hear many different perspectives and hear from people with a wide range of backgrounds. We are not making any judgments or assumptions based on this information.

I will read the questions to you or if you prefer, you can read the questions on your own.

[Read or have patient read the questions on his/her own and verbally respond to question]

Patient Background Information

1. What type of diabetes do you have?

a) Type I
b) Type II
c) Borderline diabetes
d) Not sure

2. How long have you had diabetes?

a) Less than 5 years
b) Between 5-10 years
c) Between 10-15 years
d) More than 15 years
e) Not sure

3. Do you drive yourself to your clinic appointments? (If no, who drives you)?

a) Yes
b) No, my______________________________drives me to appointments
c) No, I take public transportation, which is: _______________________
d) No, I take another type of transportation, which is: ________________

4. Are you easily able to get your clinic appointments during regular clinic hours?

a) Yes
b) No

5. What level of school have you completed?

6. We know that health information is often written in a way that is complicated and hard to understand. How often do you need to have someone help you when you read instructions, pamphlets, or other written material from your doctor or pharmacy?

a) Never
b) Rarely
c) Sometimes
d) Often
e) Always

7. What language do you speak at home? _______________

8. How well do you speak/understand English?

a) Very well (fluent)
b) Well (conversational)
c) Not well
d) Not at all

9. What is your preferred language during clinic appointments?

10. [If preferred language is not English] **Who helps you during your clinic appointments when the doctor or clinic staff does not speak your preferred language?**

11. What is your Hispanic, Latino or Spanish origin?

a) Mexican, Mexican American or Chicano
b) Puerto Rican
c) Cuban
d) Other: _____________________

Thank you so much again for your time and for this very helpful information.

Do you have any questions before we end this interview and I stop audio recording?

Introducción:

Muchas gracias por dedicar su tiempo a reunirse conmigo. Nuestro equipo de investigación de la Universidad de Wisconsin está trabajando con Access Community Health Centers para saber más sobre las experiencias de los pacientes con los controles de la vista para diabéticos y la comunicación con los proveedores de atención médica.

Esperamos utilizar los conocimientos que obtengamos de estas entrevistas para entender cómo prevenir la pérdida de visión a causa de la diabetes y descubrir los factores que facilitan o dificultan los controles de la vista para diabéticos para usted y otras personas de su comunidad.

Usted fue invitado/a a participar porque recibe atención para la diabetes en Access Community Health Centers. Espero que nuestra conversación dure, aproximadamente, una hora. Al final de nuestra conversación, le haré algunas preguntas sobre sus antecedentes.

La participación en esta entrevista es voluntaria. Su decisión de participar no afectará su atención en Access Community Health Centers. Puede detener la entrevista en cualquier momento y, si hay alguna pregunta que no quiera responder, puede decirme que la omita.

Todo lo que usted me diga hoy será confidencial. Únicamente nuestro equipo de investigación, dirigido por la doctora Maria Mora Pinzón, doctora en medicina preventiva de la Universidad de Wisconsin, y la doctora Yao Liu, oculista de la Universidad de Wisconsin, tendrán acceso a esta información. Soy un miembro del equipo de investigación que trabaja con ellas y, aunque hablaremos de temas relacionados con la salud, quiero informarle que no tengo formación médica.

La información de salud protegida (PHI) es información sobre su salud física o mental que incluye su nombre u otra información que pueda identificarlo. Para hacer este estudio, usaremos las cosas que le diga al equipo de investigación sobre su salud.

Su autorización para que los investigadores utilicen su PHI no tiene una fecha limite. Sin embargo, puede optar por retirar su autorización para que los investigadores utilicen su información de salud. Puede hacerlo en cualquier momento antes o durante su participación en la investigación. Si retira su autorización, no podrá participar en el estudio de investigación. Para retirar su autorización, deberá notificar a los investigadores.

La información recopilada en este estudio, incluida su información de salud, puede usarse o compartirse para futuras investigaciones relacionadas.

Se realizarán grabaciones de audio de la entrevista para que pueda revisar nuestra discusión más tarde y asegurarme de obtener con precisión todas las ideas y opiniones que comparte. Solo los investigadores tendrán acceso a estas grabaciones. Los investigadores o alguien contratado por los investigadores escuchará la grabación y anotará lo que dijo la gente durante la entrevista. La copia escrita se llama transcripción. La transcripción se guardará pero la grabación se destruirá. En la transcripción no se incluirá ninguna información que pueda identificarle.

Recuerde, esta entrevista es sobre sus experiencias personales. No hay respuestas "correctas" ni "incorrectas".

Si se le ocurre alguna pregunta a medida que avanzamos, no dude en hacerla en cualquier momento. ¿Tiene alguna pregunta antes de empezar?

La guía de entrevistas a pacientes: pacientes con examen de la vista

Parte I. Preguntas generales sobre los controles de la vista para diabéticos

Primero, me gustaría hacerle algunas preguntas generales sobre cómo controla su diabetes.

¿Me puede contar lo que hace a diario para manejar su diabetes?

Sugerencias:

□ *[si no tiene una rutina]* ¿Cuáles son algunas de las cosas su médico le ha comentado que son importantes que hagan las personas con diabetes?

Probablemente ha escuchado que la diabetes puede afectar los ojos y la visión.

¿Qué pueden hacer las personas con diabetes para mantener los ojos sanos?

Sugerencias:

□ ¿Conoce algún alimento, suplemento o hierba que pueda ayudar a mantener los ojos sanos?

□¿qué información ha escuchado sobre hacerse exámenes de la vista para las personas con diabetes con regularidad?

Si a alguien con diabetes le empeora la vista, ¿qué puede hacer para evitar que siga empeorando?

Sugerencias:

□ ¿Sería útil acudir a un médico/doctor de ojos?

[*Si responde si]* ¿qué podría hacer el médico/doctor de ojos para ayudar?

Ahora, me gustaría mostrarle algunas fotos. Estas fotos muestran dos formas de hacer revisiones de la vista a las personas con diabetes. La foto de arriba muestra un Examen de la Vista Tradicional, en el que un médico/doctor de ojos utiliza gotas para abrir los ojos y poder ver adentro de ellos. La foto de abajo muestra un examen fotográfico de los ojos, en el que un técnico utiliza una cámara especial para tomar fotos con el objetivo de que el médico/doctor de ojos pueda ver el interior de sus ojos después.

Algunas personas se hacen revisiones de la vista anuales como parte del cuidado de su diabetes. ¿Qué experiencias ha tenido con las revisiones de la vista para diabéticos?

Sugerencias:
□ ¿Cómo sabe cuándo ha llegado el momento de revisarse la vista por la diabetes?
□ ¿Por qué decidió hacerse una revisión de la vista?

¿Dónde aprendió sobre las revisiones de la vista para diabéticos?

Sugerencias:
□ *[Si no han recibido información sobre las revisiones de la vista.]* ¿Dónde obtuvo información sobre cómo controlar su diabetes?
□ ¿Que conoce sobre los programas de diabetes que se ofrecen en Access?
□ ¿De dónde en su comunidad le gustaría recibir información sobre los exámenes de la vista para diabéticos? *[o ¿Cómo le gustaría aprender sobre los exámenes de la vista para diabeticos?]*
□ ¿Le gustaría recibir información sobre el cuidado de su diabetes mediante un folleto/volante escrito, un video, al juntarse con un grupo, o individualmente con un educador de diabetes?

¿Se realizaría un control de la vista para diabéticos si se lo recomendara el médico que lo atiende habitualmente? ¿Por qué sí o por qué no?

Sugerencias:
□ ¿Cómo decidiría hacerse el examen de la vista?
□ Si tuviese que pagar por hacerse el examen de la vista para diabéticos, ¿cuánto estaría dispuesto a pagar?
□ Si tiene aseguranza/seguro médico ¿sabe si su seguro le cubre los exámenes de la vista para diabéticos?

Según su experiencia, ¿qué tan fácil es hacerse revisiones regulares de la vista para diabéticos en comparación con *[un ejemplo que el participante haya dado sobre lo que hace para su diabetes, preferiblemente una prueba clínica como hacerse un análisis de laboratorio para revisar la hemoglobina A1c]***?**

Sugerencias:

□ ¿Puede decirme algo más sobre por qué le resulta más fácil/difícil?

□ Usted mencionó [x] como una de las cosas que dificultan hacerse la revisión de la vista con regularidad. ¿Puede decirme algo más sobre eso? ¿Qué otra cosa podría hacerlo difícil?

□ *[si se menciona el costo o las finanzas]* Usted mencionó que el costo puede ser un problema. ¿Cómo afecta el costo en su decisión de hacerse una revisión de la vista?

¿Qué cree que ayudaría a que más personas con diabetes de su comunidad se hicieran una revisión de la vista?

[Si el paciente no se ha hecho el examen fotográfico, vaya a la página 6: Versión II. Examen Visual Clínico]

Versión I. Teleoftalmología

Se ha hecho el examen fotográfico de los ojos *[señale la foto]* ***hace [x] semanas/meses,*** **¿es correcto?**

Cuéntame sobre su experiencia, empezando por cómo te enteraste por primera vez del examen fotográfico de los ojos.
Sugerencias:
□ ¿Recuerda quién le explicó el examen?
□ ¿Qué le dijeron al respecto?
□ ¿Obtuvo toda la información que quería?
□ ¿Qué le hizo decidir hacerse el examen fotográfico de los ojos?
□ ¿Por qué cree que su médico le refirió hacerse el examen fotográfico de los ojos?

Ahora, tengo algunas preguntas sobre cómo programar una cita para el examen fotográfico de los ojos y el transporte para llegar a su cita.

¿Qué tan fácil fue programar la cita para hacerse el examen fotográfico de los ojos?
Sugerencias:
□ ¿Cuánto tiempo tuvo que esperar entre cuando programó su cita para el examen fotográfico de los ojos y cuando tuvo la cita? (por ejemplo, algunos días, semanas o meses)
□ ¿Qué tan fácil fue llegar a la clínica para el examen fotográfico de los ojos? ¿Condujo usted mismo o alguien más? ¿Cuánto tiempo tardó en llegar de su casa a la clínica?

Ahora, por favor cuénteme sobre su experiencia al recibir el examen fotográfico de los ojos.
Sugerencias:
□ ¿Aproximadamente cuánto tiempo tomó hacerse el examen fotográfico de los ojos?
□ ¿Recibió los resultados del examen fotográfico de los ojos?
□ [Si la respuesta es SÍ] ¿Qué tan rápido obtuvo los resultados? ¿Hay algo que se pueda cambiar o mejorar en la forma en que recibe los resultados?
□ [Si NO] ¿Quería recibir sus resultados del examen fotográfico de los ojos?
□ ¿Cómo le gustaría recibir los resultados del examen fotográfico de los ojos? (por ejemplo, carta por correo o llamada telefónica)

¿Como le explicaría el examen fotográfico de los ojos a un amigo o familiar?
Sugerencias:
□ ¿Estaría dispuesto a volver a hacerse el examen fotografico de los ojos? ¿Por qué?

¿Cree que hay algo que podría mejorar su experiencia con el examen fotográfico de los ojos?
Sugerencias:
□ ¿Cómo podemos hacer que el examen fotográfico de los ojos sea más accesible para otras personas en su comunidad que tienen diabetes?
□ Para su comunidad, ¿piensa que $20 es un costo razonable/apropiado para examen fotográfico de los ojos?

¿Planea hacerse otro examen de la vista para diabéticos en el futuro?
Sugerencias:
□ [Si la respuesta es SÍ] ¿Qué tan pronto y dónde podría programarlo? [verifique qué tipo de examen: examen de la vista tradicional versus prueba de la foto del ojo]
□ [Si NO] ¿Por qué no?

Veamos la imagen del examen de la vista tradicional [señale la foto]. ¿Alguna vez se ha hecho este examen en el que un oftalmólogo usa gotas para dilatar y examinar sus ojos?
Sugerencias:
□ [Si la respuesta es SÍ] Cuénteme sobre su experiencia con este examen. ¿Cómo fue?

Veamos de nuevo la imagen de los dos tipos de exámenes de los ojos. Si tuviera que elegir entre hacerse un examen de los ojos mediante un examen del ojo tradicional o un examen fotográfico de los ojos, ¿cuál preferiría? ¿Por qué?

> *Sugerencias:*
> □ ¿Cuáles podrían ser algunas de las razones por las que alguien preferiría *[la otra opción: el examen de la vista tradicional o el examen fotográfico de los ojos]*?
> □ ¿Se sentiría más cómodo haciéndose el examen fotográfico de los ojos si las fotos las revisara un médico/doctor de ojos o una computadora?

¿Hay algo más que considere importante que yo sepa sobre sus experiencias al hacerse revisiones de la vista para diabéticos?

[Vaya a la Parte II en la página 7]

Versión II. Examen Visual Clínico

No se ha hecho el examen fotográfico de los ojos *[señale la foto],* ¿es correcto?

Veamos la imagen del examen de la vista tradicional *[señale la foto]*. ¿Recuerda, aproximadamente, cuándo fue la última vez que se hizo un examen de la vista tradicional?

¿Cómo describiría el examen de la vista tradicional a un amigo o familiar?

Sugerencias:

□ ¿Volvería a hacerse un examen de la vista tradicional? ¿Por qué sí o por qué no?

□ *[EN CASO NEGATIVO]* ¿Qué tendría que cambiar para que decidiera hacerse una revisión de la vista?

¿Su seguro cubre los exámenes de la vista tradicionales? ¿Cuánto suele pagar por hacerse un examen de la vista tradicional?

Si tuviera que elegir entre hacerse un examen de la vista tradicional o un examen fotográfico de los ojos, ¿cómo decidiría qué examen hacerse? ¿Qué examen preferiría? ¿Por qué?

Sugerencias:

□ ¿Cuáles podrían ser algunas de las razones por las que alguien preferiría *[la otra opción: el examen de la vista tradicional O el examen fotográfico de los ojos]*?

□ ¿Se sentiría más cómodo haciéndose el examen fotográfico de los ojos si las fotos las revisara un médico/doctor de ojos o una computadora?

¿Hay algo más que considere importante que yo sepa sobre sus experiencias al hacerse revisiones de la vista para diabéticos?

Parte II. Preguntas relacionadas con las preferencias de comunicación

Muchas gracias por compartir su tiempo y sabiduría conmigo. Ahora nos gustaría hablar acerca del sitio en donde recibe atención médica, sus perspectivas y las experiencias que ha tenido al comunicarse con los proveedores de atención médica.

¿Cómo decidió acudir a su clínica actual donde recibe sus cuidados médicos?

Sugerencias:

□ ¿Qué tan importante es para usted encontrar un proveedor de atención médica que hable [español o su idioma preferido]?

□ ¿Lo/la ayuda su familia a decidir dónde ir para recibir atención médica? De ser así, ¿cómo lo/la ayudan?

[Si el paciente parece sentirse cómodo con el lugar en el que recibe atención médica]
Al escucharlo/la, tengo la sensación de que se siente bastante cómodo con la atención médica que recibe *[en [la clínica/organización de atención médica X] o con el/la Dr./Dra. X]*. ¿Es correcto? ¿Puede contarme sobre las cosas que lo/la hizo sentir cómodo en esa/ese [clínica/lugar]?

¿Qué podría hacer que se sintiera más cómodo al recibir atención en una clínica de la vista que no ha visitado antes?

Sugerencias:
□ ¿Cómo encuentra información sobre dónde podría ir a revisarse los ojos?
□ ¿A quién de su comunidad recurriría para que le recomendara una clínica de la vista o un médico/doctor de ojos?
□ ¿Qué podría hacer una clínica de la vista para que se sienta más cómodo/a al acudir allí?

Piense en un momento en el que haya tenido una muy buena conversación con su médico o enfermero sobre su salud. Por favor cuénteme por qué esa conversación fue tan buena experiencia.

Sugerencias:
[SI TIENEN UN EJEMPLO]
□ ¿En qué idioma hablaron? ¿Utilizó un intérprete?
□ ¿Qué le hizo sentirse más cómodo para hacer preguntas?
□ ¿Qué le hizo sentirse menos cómodo para hacer preguntas?
□ ¿Qué preguntas tenía que no se respondieron debido a un problema con la comunicación?

[SI NO TIENEN UN EJEMPLO]
Piense en una conversación con su médico o enfermero sobre su salud que podría haber sido mejor. ¿Qué podría haber hecho que la conversación fuera mejor?
□ ¿En qué idioma hablaron? ¿Utilizó un intérprete?
□ ¿Qué le hizo sentirse más cómodo para hacer preguntas?
□ ¿Qué le hizo sentirse menos cómodo para hacer preguntas?
□ ¿Qué preguntas tenía que no se respondieron debido a un problema con la comunicación?

Ahora me gustaría preguntarle sobre sus experiencias con el uso de la telemedicina. Durante la pandemia por COVID-19, algunos pacientes con diabetes han decidido tener visitas de telemedicina por video o teléfono con su médico en lugar de acudir en persona a la clínica.

¿Qué experiencia ha tenido con el uso de visitas de telemedicina por video o teléfono con alguno de sus médicos?

Sugerencias:

□ *[SI TIENE EXPERIENCIA CON LA TELEMEDICINA]* ¿Cuándo empezó a realizar este tipo de visitas?

- □ ¿Qué tan cómodo/a se siente con las visitas de telemedicina por video o teléfono con sus médicos?
- □ ¿Qué le gusta de este tipo de visitas en comparación con ver a su médico en persona?
- □ ¿Qué *no* le gusta de este tipo de visitas en comparación con ver a su médico en persona?

□ *[SI NO TIENE EXPERIENCIA CON LA TELEMEDICINA]* ¿Qué tan cómodo/a se sentiría con las visitas de telemedicina por video o teléfono con sus médicos?

- □ ¿Cómo se siente sobre este tipo de visitas en comparación con ver a su médico en persona?

[Termina la grabación]

Parte 3. Resumen y encuesta demográfica

¿Hay algo más que considere importante que yo sepa sobre las revisiones de la vista para diabéticos o la comunicación con proveedores de atención médica y que no le haya preguntado?

Muchas gracias por toda esta información tan útil. Le agradezco mucho que comparta sus experiencias conmigo. Nuestro objetivo es utilizar los conocimientos que obtengamos de estas entrevistas para mejorar los servicios de atención de la vista para las personas con diabetes de su comunidad.

Y, por último, tengo una breve lista de preguntas para obtener información de antecedentes. Esta información nos ayuda a tener una idea precisa de quiénes participan en nuestro estudio; sabemos que la comunidad es diversa y queremos asegurarnos de escuchar muchas perspectivas diferentes y oír a personas con una gran variedad de experiencias. No estamos juzgando ni haciendo suposiciones con esta información.

Le leeré las preguntas o, si lo prefiere, puede leerlas usted mismo/a.

[Lea o haga que el paciente lea las preguntas por sí mismo/a y responda verbalmente a la pregunta]

Información sobre los antecedentes del paciente

1. **¿Qué tipo de diabetes tiene?**
   a) Tipo I
   b) Tipo II
   c) Prediabetes
   d) No estoy seguro

2. **¿Hace cuánto que tiene diabetes?**
   a) Menos de 5 años
   b) Entre 5 y 10 años
   c) Entre 10 y 15 años
   d) Más de 15 años
   e) No estoy seguro

3. **¿Conduce usted mismo/a a sus citas de la vista? (De no ser así, ¿quién lo/la lleva?)**
   a) Sí
   b) No, mi_ me lleva a las citas
   c) No, voy en transporte público, que es: ________________________
   d) No, voy en otro tipo de transporte, que es: ________________

4. **¿Le resulta fácil asistir a sus citas de la vista durante el horario habitual de la clínica?**
   a) Sí
   b) No

5. **¿Qué nivel de estudios tiene?**

6. Sabemos que la información médica suele escribirse de forma complicada y difícil de entender. ¿Con qué frecuencia necesita que alguien lo ayude cuando lee instrucciones, folletos u otro material escrito de su médico o farmacia?

a) Nunca
b) Pocas veces
c) A veces
d) Con frecuencia
e) Siempre

7. ¿Qué idioma utiliza en su hogar? _______________

8. ¿Qué tan bien habla/entiende el inglés?

a) Muy bien (con fluidez)
b) Bien (conversacional)
c) No muy bien
d) Para nada

9. ¿Cuál es su idioma preferido durante las citas de la vista?

10. [Si su idioma preferido no es el inglés] **¿Quién lo/la ayuda durante sus citas de la vista cuando el médico o el personal de la clínica no hablan su idioma preferido?**

11. ¿Cuál es su origen hispano, latino o español?

a) Mexicano, estadounidense de origen mexicano o chicano
b) Puertorriqueño
c) Cubano
d) Otro: _____________________

De nuevo, muchas gracias por su tiempo y por esta información tan útil.

¿Tiene alguna pregunta antes de que terminemos esta entrevista y deje de grabar el audio?
