# Supplementary material for: Perspectives of Latinx Patients with Diabetes on Teleophthalmology, Artificial Intelligence-Based Image Interpretation, and Virtual Care: A Qualitative Study
Source: Telemed Rep. 2023 Oct 20;4(1):317–26. doi: 10.1089/tmr.2023.0045 (PMC10615055; doi:10.1089/tmr.2023.0045)
Supplement: Supplemental data [file Suppl_DataS2.pdf]

Supplement 2. Weblinks to representative examples of the pictures shown to interview participants.

A. Image of a patient obtaining a traditional dilated eye exam: https://eyepatient.net/Home/articledetail/ophthalmoscopy-4660

B. Image of a patient having their eyes photographed using teleophthalmology: https://topconhealthcare.jp/wp-content/uploads/2022/12/M000039G-4_TRC-NW400_Brochure-for-web_Global_E_2210.pdf (page 2)
